# Schizophrenia and Types of Stroke: A Mendelian Randomization Study

**DOI:** 10.1161/JAHA.123.032011

**Published:** 2024-02-29

**Authors:** Shinya Nakada, Frederick K. Ho, Carlos Celis‐Morales, Jill P. Pell

**Affiliations:** ^1^ School of Health and Wellbeing University of Glasgow Glasgow United Kingdom; ^2^ School of Cardiovascular and Metabolic Health University of Glasgow United Kingdom; ^3^ Human Performance Laboratory, Education, Physical Activity and Health Research Unit Universidad Católica del Maule Talca Chile

**Keywords:** hemorrhagic, ischemic, Mendelian randomization, schizophrenia, stroke, Cardiovascular Disease, Mental Health, Ischemic Stroke, Intracranial Hemorrhage, Cerebrovascular Disease/Stroke

## Abstract

**Background:**

Previous studies suggest an association between schizophrenia and stroke, but no studies have investigated stroke subtypes. We examined potential causal associations between schizophrenia and a range of atherosclerotic, embolic, and hemorrhagic stroke outcomes.

**Methods and Results:**

Two‐sample Mendelian randomization analyses were conducted. The summary‐level data (restricted to European ancestry) were obtained for schizophrenia and stroke: ischemic stroke, large‐artery stroke, small‐vessel stroke, cardioembolic stroke, and intracerebral hemorrhage. The associations between schizophrenia and each outcome were analyzed by an inverse variance weighting method primarily and Mendelian randomization Egger, weighted median, and weighted mode subsequently. The presence of pleiotropy was also tested by Cochran *Q* statistic, *I* ^2^ index, and Mendelian randomization Egger intercept with scatter and funnel plots. We found associations between schizophrenia and cardioembolic stroke (odds ratio [OR], 1.070 [95% CI, 1.023–1.119]) and intracerebral hemorrhage (OR, 1.089 [95% CI, 1.005–1.180]) using inverse variance weighting. Little evidence of associations with the other stroke subtypes was found. Different Mendelian randomization methods corroborated the association with cardioembolic stroke but not intracerebral hemorrhage.

**Conclusions:**

We have provided evidence of a potentially causal association between schizophrenia and cardioembolic stroke. Our findings suggest that cardiac evaluation should be considered for those with schizophrenia.

**Nonstandard Abbreviations and Acronyms**
MR: Mendelian randomization

**Clinical Perspective**

**What Is New?**

- Previous studies have suggested an association between schizophrenia and stroke, but no studies have investigated stroke subtypes systematically.
- Our findings provide evidence of a potentially causal association between schizophrenia and cardioembolic stroke.

**What Are the Clinical Implications?**

- Cardioembolic stroke accounts for 14% to 30% of ischemic strokes and is associated with more severe outcomes than atherosclerotic strokes (10‐year mortality after admission: 96% versus 84%).
- Our findings suggest that cardiac evaluation should be considered for people with schizophrenia.

Schizophrenia affects >20 million people worldwide, with a 40% increase in prevalence over the past decade.^1^, ^2^ Middle‐aged populations account for two‐thirds of cases, and it contributes to shortened life expectancy and a large public health burden.^2^, ^3^ People with schizophrenia often have metabolic abnormalities. A meta‐analysis pooling data on 25 692 people with schizophrenia reported a high prevalence of obesity, hypertension, hyperglycemia, and dyslipidemia, conferring an elevated risk of stroke on affected individuals.

Two meta‐analyses have examined an association between schizophrenia and stroke. The first meta‐analysis, conducted in 2013, identified 7 cohort studies and reported an increased risk of incident stroke for those with schizophrenia (pooled relative risk, 1.71 [95% CI, 1.19–2.46]).^4^ Subsequently, a larger meta‐analysis, in 2017, identified 14 cohort studies adjusting for covariates and reported a nearly 2‐fold higher risk of incident stroke (a pooled hazard ratio, 1.95 [95% CI, 1.41–2.70]).^5^

However, there is a paucity of evidence on associations with stroke subtypes (eg, ischemic versus hemorrhagic stroke), which is important because their risk factors and mechanisms can differ.^6^ Systematically investigating these subtypes would provide insights into the underlying biological pathways and therefore potential interventions. Furthermore, traditional observational studies are subject to unmeasured confounders between exposure and outcome variables, violating exchangeability. This limitation can be handled using instrumental variable methods.^7^, ^8^ Mendelian randomization (MR) uses genetic variants as instrumental variables to examine a causal association under specific assumptions.^9^, ^10^

We aimed to examine the potential causal associations between schizophrenia and a range of atherosclerotic, embolic, and hemorrhagic strokes using MR.

## Methods

The data that support the findings of this study are available from https://pgc.unc.edu/ and http://www.megastroke.org/ after researchers agree to comply with the conditions.

### Study Design and Data Sources

Two‐sample MR was conducted using genetic summary‐level data. The largest studies were retrieved from  the Integrative Epidemiology Unit OpenGenome Wide Association Studies (GWAS) Database Project^11^, ^12^ to investigate traits related to schizophrenia and subtypes of atherosclerotic, embolic, and hemorrhagic strokes, restricted to European populations assuming no overlap between schizophrenia and these stroke samples.

Other types of stroke (subdural hematoma and subarachnoid hemorrhage) were not included because they are different from common strokes in terms of their cause and were not our interest. Details of the contributing studies were summarized in Table S1.

The summary‐level data for schizophrenia (52 017 cases, 75 889 controls) were generated from a genome‐wide association meta‐analysis by the PCG (Psychiatric Genomics Consortium).^13^ The definition and ascertainment procedures of schizophrenia are described elsewhere.^13^ The summary‐level data for atherosclerotic and embolic strokes were generated from a genome‐wide association meta‐analysis by the MEGASTROKE consortium.^14^ Ischemic stroke (34 217 cases, 406 111 controls) was subdivided into 3 categories along with the Trial of Org 10 172 in Acute Stroke Treatment criteria: large‐vessel ischemic stroke (4373 cases, 146 392 controls), small‐vessel ischemic stroke (5386 cases, 192 662 controls), and cardioembolic ischemic stroke (7193 cases, 204 570 controls). The detailed definitions and ascertainment procedures of these strokes are summarized elsewhere.^14^ The summary‐level data for intracerebral hemorrhage (1687 cases, 201 146 controls) were generated by FinnGen.^15^ Intracerebral hemorrhage was defined as I61 based on *International Classification of Diseases, Tenth Revision* (*ICD‐10*) codes. In our analyses, single nucleotide polymorphisms (SNPs) associated with schizophrenia (*P* value <5×10^−8^) were included. After clumping (*r* ^2^<0.1 across a 1‐Mb window), SNPs that were not available in the outcome data set were replaced with proxy SNPs (*r* ^2^≥0.8). Palindromic SNPs were removed if the minor allele frequency was >0.4. The original studies obtained ethical approval and informed consent from the participants.

### Assumptions

Three core assumptions were made in conducting MR analyses: genetic variants are associated with schizophrenia (relevance assumption), independent of any potential confounders (independence assumption), and associated with stroke outcomes of interest only through schizophrenia (exclusion restriction assumption) (ie, no directional pleiotropy).^10^ Instrument association strength was assessed using the *F* statistic.^16^ All summary data were generated in people of European ancestry and adjusted for covariates (Table S1). Pleiotropy was tested in the following statistical analyses.

### Statistical Analysis

All SNP effects in summary data were obtained in log‐transformed units. For each SNP, the Wald ratio was calculated by dividing the association between SNP and stroke outcomes by the association between SNP and schizophrenia. Standard errors were estimated by the delta method. In our main analysis, these estimates were combined by a fixed‐effect inverse variance weighting meta‐analysis and expressed as odds ratios (ORs) and 95% CIs, assuming all SNPs are valid instruments.^17^ Our estimates can be interpreted as OR per 2.72‐fold multiplicative increase in the odds of the exposure.^18^ A scatter plot, Cochran *Q* test, and an *I* ^2^ index were used to test heterogeneity, and a funnel plot and MR Egger regression intercept were used to further test directional pleiotropy.^19^ In case of invalid instruments, sensitivity analyses were conducted using MR Egger regression, weighted median, and weighted mode with different assumptions. MR Egger regresses the SNP‐stroke outcome association on the SNP‐schizophrenia association allowing the intercept to be non‐0. This method gives consistent estimates even if all genetic variants are invalid, assuming there is no correlation between pleiotropic effects and SNP‐schizophrenia associations.^20^ The weighted median assumes that at least 50% of the weight comes from valid SNPs.^21^ The weighted mode assumes that the most common effect is consistent with the true causal effect.^22^ We additionally conducted multivariable MR^23^ to assess the pleiotropy due to smoking using summary‐level data on smoking initiation from the GWAS and Sequencing Consortium of Alcohol and Nicotine use.^24^ Relevance, independence, and exclusion restriction assumptions were also confirmed for multivariable MR specifically.

Any influential SNPs driving the associations between schizophrenia and stroke outcomes were identified by leave‐1‐out analysis: leaving each SNP out of the analysis alternately and repeating this for all SNPs.

Statistical analyses were conducted using the TwoSampleMR package in R version 3.5.3.

## Results

**Figure 1:**
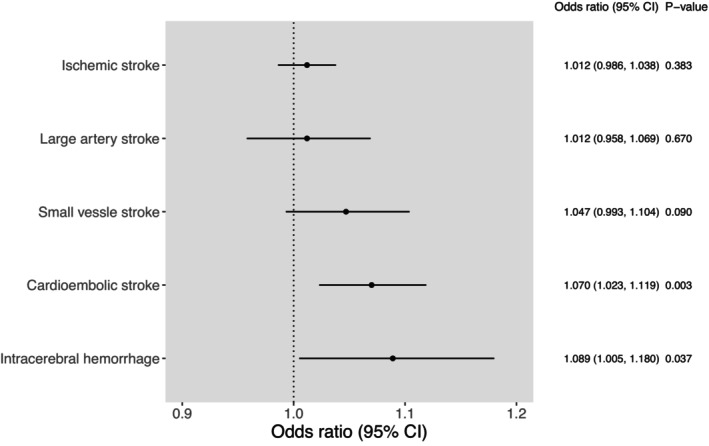
Associations between schizophrenia and stroke subtypes based on Mendelian randomization estimates. Estimates were derived using the inverse variance weighted method under a fixed‐effects model. Odds ratios are scaled per 2.72‐fold multiplicative increase in the odds of the exposure.

Although schizophrenia was not associated with ischemic stroke overall using the inverse variance weighting method, it was associated with 2 subtypes: cardioembolic stroke (OR, 1.070 [95% CI, 1.023–1.119]) and intracerebral hemorrhage (OR, 1.089 [95% CI, 1.005–1.180]) specifically (Figure 1). The association with small‐vessel stroke was only suggestive, with 95% CIs crossing the null (OR, 1.047 [95% CI, 0.993–1.104]). There was little evidence of association with large‐artery stroke (Figure 1).

**Figure 2:**
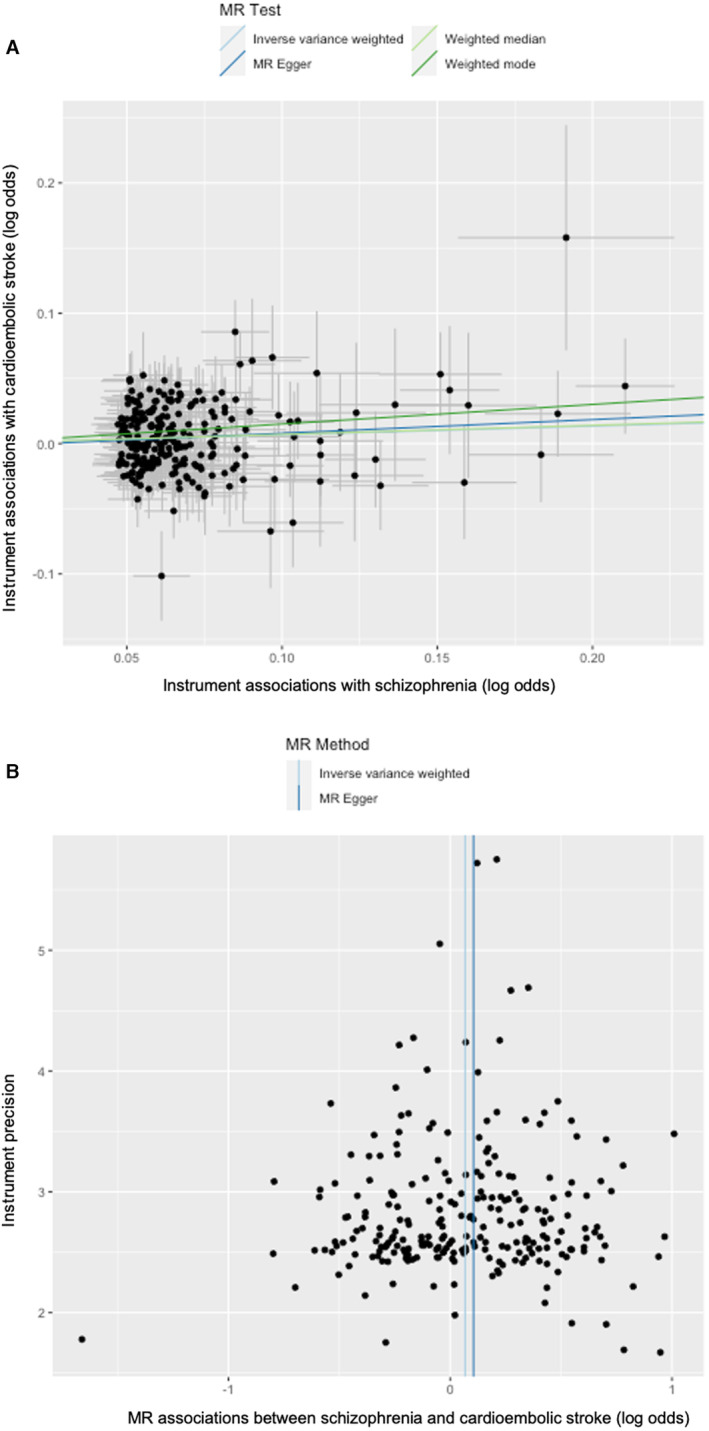
Scatterplot and funnel plot of the association between schizophrenia and cardioembolic stroke. **A**, Scatterplot of instrument associations with schizophrenia (*y* axis) and cardioembolic stroke (*x* axis) with the slopes of MR estimates. **B**, Funnel plot of instrument precision (*y* axis) and MR associations between schizophrenia and cardioembolic stroke (*x* axis). MR indicates Mendelian randomization.

The *F* statistic was 43.1 for cardioembolic stroke and 43.4 for intracerebral hemorrhage. The scatterplot of instrument associations with schizophrenia and cardioembolic stroke (Figure 2A) and Cochran *Q* test (*P*=0.267) and *I* ^2^ index (4.9) showed little evidence of heterogeneity (Table S2). The funnel plot of cardioembolic stroke was slightly asymmetrical (Figure 2B), but MR Egger showed little evidence of directional pleiotropy with a small intercept (−0.003 [95% CI, −0.014 to 0.009]) (Table 1). The sensitivity analyses using the additional 3 MR methods showed similar associations with cardioembolic stroke as the inverse variance weighting method. The weighted median gave a more precise estimate (OR, 1.073 [95% CI, 1.006–1.144]) than MR Egger (OR, 1.111 [95% CI, 0.937–1.317]) and weighted mode (OR, 1.162 [95% CI, 0.955–1.415]). After including smoking initiation in the multivariable MR, the association remained unchanged (OR, 1.063 [95% CI, 1.016–1.112]) (Table S3). The leave‐1‐out analysis did not show any single SNPs driving this association, suggesting almost even contributions of each SNP (Figure S1).

**Table 1:** Sensitivity Analyses of the Associations Between Schizophrenia and Cardioembolic Stroke and Intracerebral Hemorrhage Based on Inverse Variance Weighted, MR Egger, Weighted Median, and Weighted Mode Methods

Methods	OR	95% CI		*P* value
Ischemic stroke (247 SNPs)				
Inverse variance weighted	1.012	0.986	1.038	0.383
MR Egger*	1.019	0.924	1.124	0.709
Weighted median	1.011	0.975	1.047	0.562
Weighted mode	1.022	0.927	1.126	0.666
Large‐artery stroke (244 SNPs)				
Inverse variance weighted	1.012	0.958	1.069	0.670
MR Egger*	1.180	0.960	1.450	0.117
Weighted median	1.027	0.956	1.105	0.466
Weighted mode	1.344	0.828	1.293	0.767
Small‐vessel stroke (246 SNPs)				
Inverse variance weighted	1.047	0.993	1.104	0.090
MR Egger*	1.017	0.832	1.243	0.867
Weighted median	1.016	0.939	1.099	0.670
Weighted mode	0.954	0.792	1.148	0.616
Cardioembolic stroke (243 SNPs)				
Inverse variance weighted	1.070	1.023	1.119	0.003
MR Egger*	1.111	0.937	1.317	0.226
Weighted median	1.073	1.006	1.144	0.032
Weighted mode	1.162	0.955	1.415	0.135
Intracerebral hemorrhage (244 SNPs)				
Inverse variance weighted	1.089	1.005	1.179	0.037
MR Egger*	0.877	0.667	1.154	0.350
Weighted median	1.098	0.974	1.237	0.127
Weighted mode	1.100	0.828	1.462	0.510

ORs are scaled to per 2.72‐fold multiplicative increase in the odds of the exposure. MR indicates Mendelian randomization; OR, odds ratio; and SNPs, single nucleotide polymorphisms.

* MR Egger intercept: ischemic stroke −0.0005 (95% CI, −0.0069 to 0.0059; *P*=0.882); large‐artery stroke −0.010 (95% CI, −0.024 to 0.003; *P*=0.130); small‐vessel stroke 0.002 (95% CI, −0.011 to 0.015; *P*=0.769); cardioembolic stroke −0.003 (95% CI, −0.014 to 0.009; *P*=0.651); intracerebral stroke 0.015 (95% CI, 0.033 to −0.003; *P*=0.108).

**Figure 3:**
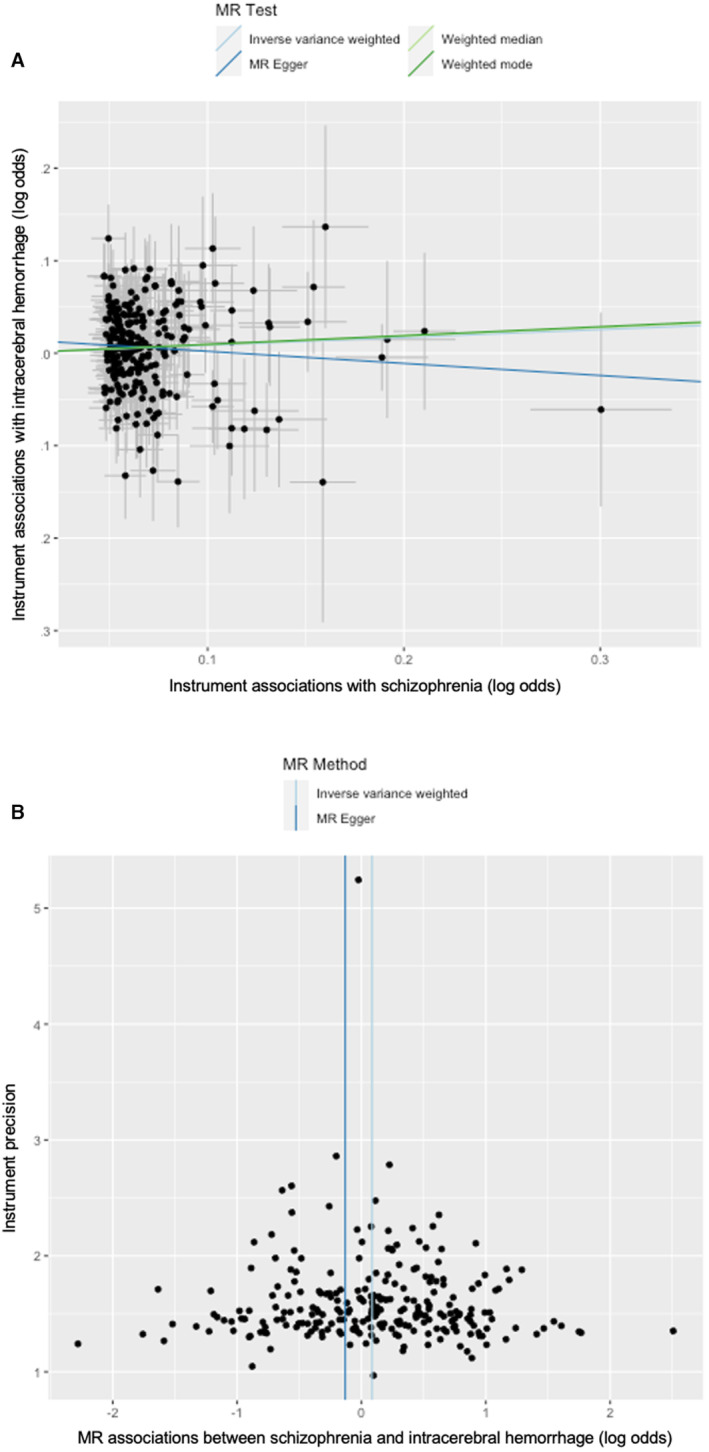
Scatterplot and funnel plot of the association between schizophrenia and intracerebral hemorrhage. **A**, Scatterplot of instrument associations with schizophrenia (*y* axis) and intracerebral hemorrhage (*x* axis) with the slopes of MR estimates. **B**, Funnel plot of instrument precision (*y* axis) and Mendelian randomization associations between schizophrenia and intracerebral hemorrhage (*x* axis). MR indicates Mendelian randomization.

Despite little evidence of heterogeneity (Figure 3A, Table S2) and directional pleiotropy (Figure 3B, Table 1) for intracerebral hemorrhage, different MR methods, and multivariable MR gave inconsistent estimate directions with large uncertainty (Table 1, Table S3). The leave‐1‐out analysis also showed 5 out of 244 SNPs driving the association with intracerebral hemorrhage (Figure S2 and Table S4).

## Discussion

### Primary Findings

This is the first study to systematically investigate potential causal associations between schizophrenia and stroke subtypes using 2‐sample MR. We have provided evidence of associations between schizophrenia and cardioembolic stroke and intracerebral hemorrhage in contrast to other stroke subtypes. However, although the estimate for cardioembolic stroke was unlikely to be due to a pleiotropic effect, that of intracerebral hemorrhage was inconclusive.

### Strengths and Limitations

By using genotypes as instrumental variables, MR overcomes the potential problem of unknown and unmeasured confounding inherit in traditional observational studies.^9^, ^10^ Therefore, although observational studies can provide evidence of association, MR studies can infer causation.^7^, ^8^ However, caution is needed in interpreting our findings. As with all MR studies, there are 3 core assumptions in our study: relevance, independence, and exclusion restriction assumptions. The relevance assumption requires instruments to be associated with schizophrenia and violation would lead to wider CIs and weak instrument bias, which amplifies the influence of confounding and pleiotropy.^25^ To assess the instrument strength, we approximated the *F* statistic to be 43.1 for cardioembolic stroke and 43.4 for intracerebral hemorrhage, which well exceeded the conventional threshold of 10.^26^ The independence assumption is untestable directly. One of the common confounders is population stratification. To mitigate this risk, we only used the summary data generated from people of European ancestry and confirmed the instrument strength. Under the 2‐sample MR setting, assuming no overlap between schizophrenia and outcome samples, the bias would be toward the null^27^ and probably explain our modest estimates for cardioembolic stroke and intracerebral hemorrhage. The exclusion restriction assumption is also untestable, but we found little evidence of directional pleiotropy for cardioembolic stroke, subsequently corroborated by different MR methods and leave‐1‐out analysis. Some studies suggest a shared genetic background of schizophrenia and smoking^28^, ^29^ and a likely causal association of smoking with schizophrenia,^30^, ^31^, ^32^, ^33^ threatening this exclusion restriction assumption. Nonetheless, we found that the association between schizophrenia and cardioembolic stroke remained unchanged even after considering smoking initiation. Also, we treated schizophrenia as binary exposure due to lack of data on and understanding of the clinical significance of preclinical psychotic symptoms.^34^ Although this may pose another risk of pleiotropy, our results would still inform the test of the causal null hypothesis.

An additional assumption is required to infer an average causal effect. The homogeneity assumption requires the effect of schizophrenia on stroke outcomes to be the same in everyone, which is implausible. Therefore, we replaced this with a monotonicity assumption that the increasing number of risk alleles does not lower the likelihood of exposure for any individuals, allowing effect estimates to be the average causal effect only in the genetic compliers, a subgroup of the population.^18^ Because of our limited data, we could not test this assumption. Although violation obscures the interpretation of the effect estimate, it would still inform of a nonnull effect. The last limitation is winner's curse, which overestimates the associations between SNPs and a trait. Our SNP selection, based on a *P* value threshold <5×10^−8^, could overestimate the effects of SNPs on schizophrenia and the denominator of the Wald ratio accordingly. In our 2‐sample MR setting, assuming no overlap between schizophrenia and outcome samples, this would bias our estimates toward the null,^35^ partly explaining our modest estimates of cardioembolic stroke and intracerebral hemorrhage and null results for other stroke subtypes. Finally, care is needed in applying our findings to non‐European (eg, Asian) populations, because we restricted the analyses to European ancestry.

### Comparison With Prior Studies

Our findings of associations between schizophrenia and cardioembolic stroke and intracerebral hemorrhage are generally consistent with evidence from previous observational studies suggesting an association between schizophrenia and stroke.^4^, ^5^ However, few studies have examined the association between schizophrenia and cardioembolic stroke specifically. A hypothetical explanation for our finding is the use of second‐generation antipsychotic drugs. One case–crossover study reported an association between second‐generation antipsychotic drugs and ischemic stroke (including cardioembolic stroke) among patients with schizophrenia, although the mechanism remains unclear.^36^ Another explanation is cardiac problems (eg, atrial fibrillation and heart failure),^37^ which are associated with schizophrenia. A prospective study reported a 2‐fold higher risk of atrial fibrillation among those with schizophrenia compared with those without the condition.^38^ It was also found that patients with schizophrenia were less likely to be prescribed anticoagulants.^39^ These could explain the association observed with cardioembolic stroke specifically but not ischemic stroke overall, because atrial fibrillation is only a risk factor of the former. Similarly, an MR study and meta‐analyses reported an association with heart failure.^5^, ^40^ Some reports have suggested increased sympathetic nerve and decreased parasympathetic nerve activities among those with schizophrenia, which could overload cardiac functions.^41^

We found little evidence of an association with other types of ischemic stroke. Although previous studies investigating subtypes of stroke are scarce, our findings in relation to ischemic stroke are consistent with a previous MR study that reported no association between schizophrenia and other atherosclerotic vascular problems such as coronary heart disease.^40^

Previous evidence for intracerebral hemorrhage is also limited. One MR study has suggested a lack of association between schizophrenia and intracerebral hemorrhage,^42^ and another study found little association between second‐generation antipsychotic drugs and intracerebral hemorrhage among patients with schizophrenia.^36^ If there exists an explanation in favor of our finding, it may be the more common use of anticoagulant drugs due to cardiac problems (eg, atrial fibrillation) among patients with schizophrenia. However, a recent Danish study has pointed out that patients with schizophrenia and atrial fibrillation are less likely to initiate and adhere to oral anticoagulation therapy.^39^ Further studies are needed to determine whether there is a causal association between schizophrenia and intracerebral hemorrhage and, if so, its underlying mechanism.

### Implications

Our study has provided evidence of a causal association between schizophrenia and cardioembolic stroke. Given the increasing number of people with schizophrenia worldwide, the prevention of stroke in this subpopulation is of great importance. Cardioembolic stroke accounts for 14% to 30% of ischemic strokes^43^ and is associated with more severe outcomes than atherosclerotic strokes (10‐year mortality after admission: 96% versus 84%),^44^ and therefore early prevention plays a key role. Our findings suggest that cardiac evaluation should be considered for those with schizophrenia.

## Conclusions

We systematically investigated the potential causal associations between schizophrenia and stroke subtypes using 2‐sample MR. We found evidence of an association between schizophrenia and cardioembolic stroke. Our findings could raise clinical awareness of early cardiac evaluation and treatments for those with schizophrenia.

## Sources of Funding

S.N. is supported by a PhD studentship award from the Medical Research Council (MR/N013166/1‐LGH/MS/MED2525).

## Disclosures

None.

## Supporting information

Data S1
Tables S1–S4
Figures S1–S2
