# Supplementary material for: Schizophrenia and Types of Stroke: A Mendelian Randomization Study
Source: J Am Heart Assoc. 2024 Feb 29;13(5):e032011. doi: 10.1161/JAHA.123.032011 (PMC10944050; doi:10.1161/JAHA.123.032011)

Supplemental Material

MEGASTROKE CONSORTIUM

Rainer Malik 1, Ganesh Chauhan 2, Matthew Traylor 3, Muralidharan Sargurupremraj 4,5, Yukinori Okada 6,7,8, Aniket Mishra 4,5, Loes Rutten-Jacobs 3, Anne-Katrin Giese 9, Sander W van der Laan 10, Solveig Gretarsdottir 11, Christopher D Anderson 12,13,14,14, Michael Chong 15, Hieab HH Adams 16,17, Tetsuro Ago 18, Peter Almgren 19, Philippe Amouyel 20,21, Hakan Ay 22,13, Traci M Bartz 23, Oscar R Benavente 24, Steve Bevan 25, Giorgio B Boncoraglio 26, Robert D Brown, Jr. 27, Adam S Butterworth 28,29, Caty Carrera 30,31, Cara L Carty 32,33, Daniel I Chasman 34,35, Wei-Min Chen 36, John W Cole 37, Adolfo Correa 38, Ioana Cotlarciuc 39, Carlos Cruchaga 40,41, John Danesh 28,42,43,44, Paul IW de Bakker 45,46, Anita L DeStefano 47,48, Marcel den Hoed 49, Qing Duan 50, Stefan T Engelter 51,52, Guido J Falcone 53,54, Rebecca F Gottesman 55, Raji P Grewal 56, Vilmundur Gudnason 57,58, Stefan Gustafsson 59, Jeffrey Haessler 60, Tamara B Harris 61, Ahamad Hassan 62, Aki S Havulinna 63,64, Susan R Heckbert 65, Elizabeth G Holliday 66,67, George Howard 68, Fang-Chi Hsu 69, Hyacinth I Hyacinth 70, M Arfan Ikram 16, Erik Ingelsson 71,72, Marguerite R Irvin 73, Xueqiu Jian 74, Jordi Jiménez-Conde 75, Julie A Johnson 76,77, J Wouter Jukema 78, Masahiro Kanai 6,7,79, Keith L Keene 80,81, Brett M Kissela 82, Dawn O Kleindorfer 82, Charles Kooperberg 60, Michiaki Kubo 83, Leslie A Lange 84, Carl D Langefeld 85, Claudia Langenberg 86, Lenore J Launer 87, Jin-Moo Lee 88, Robin Lemmens 89,90, Didier Leys 91, Cathryn M Lewis 92,93, Wei-Yu Lin 28,94, Arne G Lindgren 95,96, Erik Lorentzen 97, Patrik K Magnusson 98, Jane Maguire 99, Ani Manichaikul 36, Patrick F McArdle 100, James F Meschia 101, Braxton D Mitchell 100,102, Thomas H Mosley 103,104, Michael A Nalls 105,106, Toshiharu Ninomiya 107, Martin J O'Donnell 15,108, Bruce M Psaty 109,110,111,112, Sara L Pulit 113,45, Kristiina Rannikmäe 114,115, Alexander P Reiner 65,116, Kathryn M Rexrode 117, Kenneth Rice 118, Stephen S Rich 36, Paul M Ridker 34,35, Natalia S Rost 9,13, Peter M Rothwell 119, Jerome I Rotter 120,121, Tatjana Rundek 122, Ralph L Sacco 122, Saori Sakaue 7,123, Michele M Sale 124, Veikko Salomaa 63, Bishwa R Sapkota 125, Reinhold Schmidt 126, Carsten O Schmidt 127, Ulf Schminke 128, Pankaj Sharma 39, Agnieszka Slowik 129, Cathie LM Sudlow 114,115, Christian Tanislav 130, Turgut Tatlisumak 131,132, Kent D Taylor 120,121, Vincent NS Thijs 133,134, Gudmar Thorleifsson 11, Unnur Thorsteinsdottir 11, Steffen Tiedt 1, Stella Trompet 135, Christophe Tzourio 5,136,137, Cornelia M van Duijn 138,139, Matthew Walters 140, Nicholas J Wareham 86, Sylvia Wassertheil-Smoller 141, James G Wilson 142, Kerri L Wiggins 109, Qiong Yang 47, Salim Yusuf 15, Najaf Amin 16, Hugo S Aparicio 185,48, Donna K Arnett 186, John Attia 187, Alexa S Beiser 47,48, Claudine Berr 188, Julie E Buring 34,35, Mariana Bustamante 189, Valeria Caso 190, Yu-Ching Cheng 191, Seung Hoan Choi 192,48, Ayesha Chowhan 185,48, Natalia Cullell 31, Jean-François Dartigues 193,194, Hossein Delavaran 95,96, Pilar Delgado 195, Marcus Dörr 196,197, Gunnar Engström 19, Ian Ford 198, Wander S Gurpreet 199, Anders Hamsten 200,201, Laura Heitsch 202, Atsushi Hozawa 203, Laura Ibanez 204, Andreea Ilinca 95,96, Martin Ingelsson 205, Motoki Iwasaki 206, Rebecca D Jackson 207, Katarina Jood 208, Pekka Jousilahti 63, Sara Kaffashian 4,5, Lalit Kalra 209, Masahiro Kamouchi 210, Takanari Kitazono 211, Olafur Kjartansson 212, Manja Kloss 213, Peter J Koudstaal 214, Jerzy Krupinski 215, Daniel L Labovitz 216, Cathy C Laurie 118, Christopher R Levi 217, Linxin Li 218, Lars Lind 219, Cecilia M Lindgren 220,221, Vasileios Lioutas 222,48, Yong Mei Liu 223, Oscar L Lopez 224, Hirata Makoto 225, Nicolas Martinez-Majander 172, Koichi Matsuda 225, Naoko Minegishi 203, Joan Montaner 226, Andrew P Morris 227,228, Elena Muiño 31, Martina Müller-Nurasyid 229,230,231, Bo Norrving 95,96, Soichi Ogishima 203, Eugenio A Parati 232, Leema Reddy Peddareddygari 56, Nancy L Pedersen 98,233, Joanna Pera 129, Markus Perola 63,234, Alessandro Pezzini 235, Silvana Pileggi 236, Raquel Rabionet 237, Iolanda Riba-Llena 30,

Marta Ribasés 238, Jose R Romero 185,48, Jaume Roquer 239,240, Anthony G Rudd 241,242, Antti-Pekka Sarin 243,244, Ralhan Sarju 199, Chloe Sarnowski 47,48, Makoto Sasaki 245, Claudia L Satizabal 185,48, Mamoru Satoh 245, Naveed Sattar 246, Norie Sawada 206, Gerli Sibolt 172, Ásgeir Sigurdsson 247, Albert Smith 248, Kenji Sobue 245, Carolina Soriano-Tárraga 240, Tara Stanne 249, O Colin Stine 250, David J Stott 251, Konstantin Strauch 229,252, Takako Takai 203, Hideo Tanaka 253,254, Kozo Tanno 245, Alexander Teumer 255, Liisa Tomppo 172, Nuria P Torres-Aguila 31, Emmanuel Touze 256,257, Shoichiro Tsugane 206, Andre G Uitterlinden 258, Einar M Valdimarsson 259, Sven J van der Lee 16, Henry Völzke 255, Kenji Wakai 253, David Weir 260, Stephen R Williams 261, Charles DA Wolfe 241,242, Quenna Wong 118, Huichun Xu 191, Taiki Yamaji 206, Dharambir K Sanghera 125,169,170, Olle Melander 19, Christina Jern 171, Daniel Strbian 172,173, Israel Fernandez-Cadenas 31,30, W T Longstreth, Jr 174,65, Arndt Rolfs 175, Jun Hata 107, Daniel Woo 82, Jonathan Rosand 12,13,14, Guillaume Pare 15, Jemma C Hopewell 176, Danish Saleheen 177, Kari Stefansson 11,178, Bradford B Worrall 179, Steven J Kittner 37, Sudha Seshadri 180,48, Myriam Fornage 74,181, Hugh S Markus 3, Joanna MM Howson 28, Yoichiro Kamatani 6,182, Stephanie Debette 4,5, Martin Dichgans 1,183,184

1 Institute for Stroke and Dementia Research (ISD), University Hospital, LMU Munich, Munich, Germany
2 Centre for Brain Research, Indian Institute of Science, Bangalore, India
3 Stroke Research Group, Division of Clinical Neurosciences, University of Cambridge, UK
4 INSERM U1219 Bordeaux Population Health Research Center, Bordeaux, France
5 University of Bordeaux, Bordeaux, France
6 Laboratory for Statistical Analysis, RIKEN Center for Integrative Medical Sciences, Yokohama, Japan
7 Department of Statistical Genetics, Osaka University Graduate School of Medicine, Osaka, Japan
8 Laboratory of Statistical Immunology, Immunology Frontier Research Center (WPI-IFReC), Osaka University, Suita, Japan.
9 Department of Neurology, Massachusetts General Hospital, Harvard Medical School, Boston, MA, USA
10 Laboratory of Experimental Cardiology, Division of Heart and Lungs, University Medical Center Utrecht, University of Utrecht, Utrecht,Netherlands
11 deCODE genetics/AMGEN inc, Reykjavik, Iceland
12 Center for Genomic Medicine, Massachusetts General Hospital (MGH), Boston, MA, USA
13 J. Philip Kistler Stroke Research Center, Department of Neurology, MGH, Boston, MA, USA
14 Program in Medical and Population Genetics, Broad Institute, Cambridge, MA, USA
15 Population Health Research Institute, McMaster University, Hamilton, Canada
16 Department of Epidemiology, Erasmus University Medical Center, Rotterdam, Netherlands
17 Department of Radiology and Nuclear Medicine, Erasmus University Medical Center, Rotterdam, Netherlands
18 Department of Medicine and Clinical Science, Graduate School of Medical Sciences, Kyushu University, Fukuoka, Japan
19 Department of Clinical Sciences, Lund University, Malmö, Sweden
20 Univ. Lille, Inserm, Institut Pasteur de Lille, LabEx DISTALZ-UMR1167, Risk factors and molecular determinants of aging-related diseases, F-59000 Lille, France

21 Centre Hosp. Univ Lille, Epidemiology and Public Health Department, F-59000 Lille, France
22 AA Martinos Center for Biomedical Imaging, Department of Radiology, Massachusetts General Hospital, Harvard Medical School, Boston, MA, USA
23 Cardiovascular Health Research Unit, Departments of Biostatistics and Medicine, University of Washington, Seattle, WA, USA
24 Division of Neurology, Faculty of Medicine, Brain Research Center, University of British Columbia, Vancouver, Canada
25 School of Life Science, University of Lincoln, Lincoln, UK
26 Department of Cerebrovascular Diseases, Fondazione IRCCS Istituto Neurologico "Carlo Besta", Milano, Italy
27 Department of Neurology, Mayo Clinic Rochester, Rochester, MN, USA
28 MRC/BHF Cardiovascular Epidemiology Unit, Department of Public Health and Primary Care, University of Cambridge, Cambridge, UK
29 The National Institute for Health Research Blood and Transplant Research Unit in Donor Health and Genomics, University of Cambridge, UK
30 Neurovascular Research Laboratory, Vall d'Hebron Institut of Research, Neurology and Medicine Departments-Universitat Autònoma de Barcelona, Vall d'Hebrón Hospital, Barcelona, Spain
31 Stroke Pharmacogenomics and Genetics, Fundacio Docència i Recerca MutuaTerrassa, Terrassa, Spain
32 Children's Research Institute, Children's National Medical Center, Washington, DC, USA
33 Center for Translational Science, George Washington University, Washington, DC, USA
34 Division of Preventive Medicine, Brigham and Women's Hospital, Boston, MA, USA
35 Harvard Medical School, Boston, MA, USA
36 Center for Public Health Genomics, Department of Public Health Sciences, University of Virginia, Charlottesville, VA, USA
37 Department of Neurology, University of Maryland School of Medicine and Baltimore VAMC, Baltimore, MD, USA
38 Departments of Medicine, Pediatrics and Population Health Science, University of Mississippi Medical Center, Jackson, MS, USA
39 Institute of Cardiovascular Research, Royal Holloway University of London, UK & Ashford and St Peters Hospital, Surrey UK
40 Department of Psychiatry,The Hope Center Program on Protein Aggregation and Neurodegeneration (HPAN),Washington University, School of Medicine, St. Louis, MO, USA
41 Department of Developmental Biology, Washington University School of Medicine, St. Louis, MO, USA
42 NIHR Blood and Transplant Research Unit in Donor Health and Genomics, Department of Public Health and Primary Care, University of Cambridge, Cambridge, UK
43 Wellcome Trust Sanger Institute, Wellcome Trust Genome Campus, Hinxton, Cambridge, UK
44 British Heart Foundation, Cambridge Centre of Excellence, Department of Medicine, University of Cambridge, Cambridge, UK
45 Department of Medical Genetics, University Medical Center Utrecht, Utrecht, Netherlands
46 Department of Epidemiology, Julius Center for Health Sciences and Primary Care, University Medical Center Utrecht, Utrecht, Netherlands
47 Boston University School of Public Health, Boston, MA, USA
48 Framingham Heart Study, Framingham, MA, USA

49 Department of Immunology, Genetics and Pathology and Science for Life Laboratory, Uppsala University, Uppsala, Sweden
50 Department of Genetics, University of North Carolina, Chapel Hill, NC, USA
51 Department of Neurology and Stroke Center, Basel University Hospital, Switzerland
52 Neurorehabilitation Unit, University and University Center for Medicine of Aging and Rehabilitation Basel, Felix Platter Hospital, Basel, Switzerland
53 Department of Neurology, Yale University School of Medicine, New Haven, CT, USA
54 Program in Medical and Population Genetics, The Broad Institute of Harvard and MIT, Cambridge, MA, USA
55 Department of Neurology, Johns Hopkins University School of Medicine, Baltimore, MD, USA
56 Neuroscience Institute, SF Medical Center, Trenton, NJ, USA
57 Icelandic Heart Association Research Institute, Kopavogur, Iceland
58 University of Iceland, Faculty of Medicine, Reykjavik, Iceland
59 Department of Medical Sciences, Molecular Epidemiology and Science for Life Laboratory, Uppsala University, Uppsala, Sweden
60 Division of Public Health Sciences, Fred Hutchinson Cancer Research Center, Seattle, WA, USA
61 Laboratory of Epidemiology and Population Science, National Institute on Aging, National Institutes of Health, Bethesda, MD, USA
62 Department of Neurology, Leeds General Infirmary, Leeds Teaching Hospitals NHS Trust, Leeds, UK
63 National Institute for Health and Welfare, Helsinki, Finland
64 FIMM - Institute for Molecular Medicine Finland, Helsinki, Finland
65 Department of Epidemiology, University of Washington, Seattle, WA, USA
66 Public Health Stream, Hunter Medical Research Institute, New Lambton, Australia
67 Faculty of Health and Medicine, University of Newcastle, Newcastle, Australia
68 School of Public Health, University of Alabama at Birmingham, Birmingham, AL, USA
69 Department of Biostatistical Sciences, Wake Forest School of Medicine, Winston-Salem, NC, USA
70 Aflac Cancer and Blood Disorder Center, Department of Pediatrics, Emory University School of Medicine, Atlanta, GA, USA
71 Department of Medicine, Division of Cardiovascular Medicine, Stanford University School of Medicine, CA, USA
72 Department of Medical Sciences, Molecular Epidemiology and Science for Life Laboratory, Uppsala University, Uppsala, Sweden
73 Epidemiology, School of Public Health, University of Alabama at Birmingham, USA
74 Brown Foundation Institute of Molecular Medicine, University of Texas Health Science Center at Houston, Houston, TX, USA
75 Neurovascular Research Group (NEUVAS), Neurology Department, Institut Hospital del Mar d'Investigació Mèdica, Universitat Autònoma de Barcelona, Barcelona, Spain
76 Department of Pharmacotherapy and Translational Research and Center for Pharmacogenomics, University of Florida, College of Pharmacy, Gainesville, FL, USA
77 Division of Cardiovascular Medicine, College of Medicine, University of Florida, Gainesville, FL, USA
78 Department of Cardiology, Leiden University Medical Center, Leiden, the Netherlands
79 Program in Bioinformatics and Integrative Genomics, Harvard Medical School, Boston, MA, USA
80 Department of Biology, East Carolina University, Greenville, NC, USA
81 Center for Health Disparities, East Carolina University, Greenville, NC, USA

82 University of Cincinnati College of Medicine, Cincinnati, OH, USA
83 RIKEN Center for Integrative Medical Sciences, Yokohama, Japan
84 Department of Medicine, University of Colorado Denver, Anschutz Medical Campus, Aurora, CO, USA
85 Center for Public Health Genomics and Department of Biostatistical Sciences, Wake Forest School of Medicine, Winston-Salem, NC, USA
86 MRC Epidemiology Unit, University of Cambridge School of Clinical Medicine, Institute of Metabolic Science, Cambridge Biomedical Campus, Cambridge, UK
87 Intramural Research Program, National Institute on Aging, National Institutes of Health, Bethesda, MD, USA
88 Department of Neurology, Radiology, and Biomedical Engineering, Washington University School of Medicine, St. Louis, MO, USA
89 KU Leuven – University of Leuven, Department of Neurosciences, Experimental Neurology, Leuven, Belgium
90 VIB Center for Brain & Disease Research, University Hospitals Leuven, Department of Neurology, Leuven, Belgium
91 Univ.-Lille, INSERM U 1171. CHU Lille. Lille, France
92 Department of Medical and Molecular Genetics, King's College London, London, UK
93 SGDP Centre, Institute of Psychiatry, Psychology & Neuroscience, King's College London, London, UK
94 Northern Institute for Cancer Research, Paul O'Gorman Building, Newcastle University, Newcastle, UK
95 Department of Clinical Sciences Lund, Neurology, Lund University, Lund, Sweden
96 Department of Neurology and Rehabilitation Medicine, Skåne University Hospital, Lund, Sweden
97 Bioinformatics Core Facility, University of Gothenburg, Gothenburg, Sweden
98 Department of Medical Epidemiology and Biostatistics, Karolinska Institutet, Stockholm, Sweden
99 University of Technology Sydney, Faculty of Health, Ultimo, Australia
100 Department of Medicine, University of Maryland School of Medicine, MD, USA
101 Department of Neurology, Mayo Clinic, Jacksonville, FL, USA
102 Geriatrics Research and Education Clinical Center, Baltimore Veterans Administration Medical Center, Baltimore, MD, USA
103 Division of Geriatrics, School of Medicine, University of Mississippi Medical Center, Jackson, MS, USA
104 Memory Impairment and Neurodegenerative Dementia Center, University of Mississippi Medical Center, Jackson, MS, USA
105 Laboratory of Neurogenetics, National Institute on Aging, National institutes of Health, Bethesda, MD, USA
106 Data Tecnica International, Glen Echo MD, USA
107 Department of Epidemiology and Public Health, Graduate School of Medical Sciences, Kyushu University, Fukuoka, Japan
108 Clinical Research Facility, Department of Medicine, NUI Galway, Galway, Ireland
109 Cardiovascular Health Research Unit, Department of Medicine, University of Washington, Seattle, WA, USA
110 Department of Epidemiology, University of Washington, Seattle, WA
111 Department of Health Services, University of Washington, Seattle, WA, USA
112 Kaiser Permanente Washington Health Research Institute, Seattle, WA, USA
113 Brain Center Rudolf Magnus, Department of Neurology, University Medical Center Utrecht, Utrecht, The Netherlands

114 Usher Institute of Population Health Sciences and Informatics, University of Edinburgh, Edinburgh, UK
115 Centre for Clinical Brain Sciences, University of Edinburgh, Edinburgh, UK
116 Fred Hutchinson Cancer Research Center, University of Washington, Seattle, WA, USA
117 Department of Medicine, Brigham and Women's Hospital, Boston, MA, USA
118 Department of Biostatistics, University of Washington, Seattle, WA, USA
119 Nuffield Department of Clinical Neurosciences, University of Oxford, UK
120 Institute for Translational Genomics and Population Sciences, Los Angeles Biomedical Research Institute at Harbor-UCLA Medical Center, Torrance, CA, USA
121 Division of Genomic Outcomes, Department of Pediatrics, Harbor-UCLA Medical Center, Torrance, CA, USA
122 Department of Neurology, Miller School of Medicine, University of Miami, Miami, FL, USA
123 Department of Allergy and Rheumatology, Graduate School of Medicine, the University of Tokyo, Tokyo, Japan
124 Center for Public Health Genomics, University of Virginia, Charlottesville, VA, USA
125 Department of Pediatrics, College of Medicine, University of Oklahoma Health Sciences Center, Oklahoma City, OK, USA
126 Department of Neurology, Medical University of Graz, Graz, Austria
127 University Medicine Greifswald, Institute for Community Medicine, SHIP-KEF, Greifswald, Germany
128 University Medicine Greifswald, Department of Neurology, Greifswald, Germany
129 Department of Neurology, Jagiellonian University, Krakow, Poland
130 Department of Neurology, Justus Liebig University, Giessen, Germany
131 Department of Clinical Neurosciences/Neurology, Institute of Neuroscience and Physiology, Sahlgrenska Academy at University of Gothenburg, Gothenburg, Sweden
132 Sahlgrenska University Hospital, Gothenburg, Sweden
133 Stroke Division, Florey Institute of Neuroscience and Mental Health, University of Melbourne, Heidelberg, Australia
134 Austin Health, Department of Neurology, Heidelberg, Australia
135 Department of Internal Medicine, Section Gerontology and Geriatrics, Leiden University Medical Center, Leiden, the Netherlands
136 INSERM U1219, Bordeaux, France
137 Department of Public Health, Bordeaux University Hospital, Bordeaux, France
138 Genetic Epidemiology Unit, Department of Epidemiology, Erasmus University Medical Center Rotterdam, Netherlands
139 Center for Medical Systems Biology, Leiden, Netherlands
140 School of Medicine, Dentistry and Nursing at the University of Glasgow, Glasgow, UK
141 Department of Epidemiology and Population Health, Albert Einstein College of Medicine, NY, USA
142 Department of Physiology and Biophysics, University of Mississippi Medical Center, Jackson, MS, USA
143 A full list of members and affiliations appears in the Supplementary Note
144 Department of Human Genetics, McGill University, Montreal, Canada
145 Department of Pathophysiology, Institute of Biomedicine and Translation Medicine, University of Tartu, Tartu, Estonia
146 Department of Cardiac Surgery, Tartu University Hospital, Tartu, Estonia
147 Clinical Gene Networks AB,Stockholm, Sweden
148 Department of Genetics and Genomic Sciences, The Icahn Institute for Genomics and Multiscale Biology Icahn School of Medicine at Mount Sinai, New York, NY , USA

149 Department of Pathophysiology, Institute of Biomedicine and Translation Medicine, University of Tartu, Biomeedikum, Tartu, Estonia
150 Integrated Cardio Metabolic Centre, Department of Medicine, Karolinska Institutet, Karolinska Universitetssjukhuset, Huddinge, Sweden.
151 Clinical Gene Networks AB, Stockholm, Sweden
152 Sorbonne Universités, UPMC Univ. Paris 06, INSERM, UMR_S 1166, Team Genomics & Pathophysiology of Cardiovascular Diseases, Paris, France
153 ICAN Institute for Cardiometabolism and Nutrition, Paris, France
154 Department of Biomedical Engineering, University of Virginia, Charlottesville, VA, USA
155 Group Health Research Institute, Group Health Cooperative, Seattle, WA, USA
156 Seattle Epidemiologic Research and Information Center, VA Office of Research and Development, Seattle, WA, USA
157 Cardiovascular Research Center, Massachusetts General Hospital, Boston, MA, USA
158 Department of Medical Research, Bærum Hospital, Vestre Viken Hospital Trust, Gjettum, Norway
159 Saw Swee Hock School of Public Health, National University of Singapore and National University Health System, Singapore
160 National Heart and Lung Institute, Imperial College London, London, UK
161 Department of Gene Diagnostics and Therapeutics, Research Institute, National Center for Global Health and Medicine, Tokyo, Japan
162 Department of Epidemiology, Tulane University School of Public Health and Tropical Medicine, New Orleans, LA, USA
163 Department of Cardiology,University Medical Center Groningen, University of Groningen, Netherlands
164 MRC-PHE Centre for Environment and Health, School of Public Health, Department of Epidemiology and Biostatistics, Imperial College London, London, UK
165 Department of Epidemiology and Biostatistics, Imperial College London, London, UK
166 Department of Cardiology, Ealing Hospital NHS Trust, Southall, UK
167 National Heart, Lung and Blood Research Institute, Division of Intramural Research, Population Sciences Branch, Framingham, MA, USA
168 A full list of members and affiliations appears at the end of the manuscript
169 Department of Phamaceutical Sciences, Collge of Pharmacy, University of Oklahoma Health Sciences Center, Oklahoma City, OK, USA
170 Oklahoma Center for Neuroscience, Oklahoma City, OK, USA
171 Department of Pathology and Genetics, Institute of Biomedicine, The Sahlgrenska Academy at University of Gothenburg, Gothenburg, Sweden
172 Department of Neurology, Helsinki University Hospital, Helsinki, Finland
173 Clinical Neurosciences, Neurology, University of Helsinki, Helsinki, Finland
174 Department of Neurology, University of Washington, Seattle, WA, USA
175 Albrecht Kossel Institute, University Clinic of Rostock, Rostock, Germany
176 Clinical Trial Service Unit and Epidemiological Studies Unit, Nuffield Department of Population Health, University of Oxford, Oxford, UK
177 Department of Genetics, Perelman School of Medicine, University of Pennsylvania, PA, USA
178 Faculty of Medicine, University of Iceland, Reykjavik, Iceland
179 Departments of Neurology and Public Health Sciences, University of Virginia School of Medicine, Charlottesville, VA, USA
180 Department of Neurology, Boston University School of Medicine, Boston, MA, USA
181 Human Genetics Center, University of Texas Health Science Center at Houston, Houston, TX, USA

182 Center for Genomic Medicine, Kyoto University Graduate School of Medicine, Kyoto, Japan
183 Munich Cluster for Systems Neurology (SyNergy), Munich, Germany
184 German Center for Neurodegenerative Diseases (DZNE), Munich, Germany
185 Boston University School of Medicine, Boston, MA, USA
186 University of Kentucky College of Public Health, Lexington, KY, USA
187 University of Newcastle and Hunter Medical Research Institute, New Lambton, Australia
188 Univ. Montpellier, Inserm, U1061, Montpellier, France
189 Centre for Research in Environmental Epidemiology, Barcelona, Spain
190 Department of Neurology, Università degli Studi di Perugia, Umbria, Italy
191 Department of Medicine, University of Maryland School of Medicine, Baltimore, MD, USA
192 Broad Institute, Cambridge, MA, USA
193 Univ. Bordeaux, Inserm, Bordeaux Population Health Research Center, UMR 1219, Bordeaux, France
194 Bordeaux University Hospital, Department of Neurology, Memory Clinic, Bordeaux, France
195 Neurovascular Research Laboratory. Vall d'Hebron Institut of Research, Neurology and Medicine Departments-Universitat Autònoma de Barcelona. Vall d'Hebrón Hospital, Barcelona, Spain
196 University Medicine Greifswald, Department of Internal Medicine B, Greifswald, Germany
197 DZHK, Greifswald, Germany
198 Robertson Center for Biostatistics, University of Glasgow, Glasgow, UK
199 Hero DMC Heart Institute, Dayanand Medical College & Hospital, Ludhiana, India
200 Atherosclerosis Research Unit, Department of Medicine Solna, Karolinska Institutet, Stockholm, Sweden
201 Karolinska Institutet, Stockholm, Sweden
202 Division of Emergency Medicine, and Department of Neurology, Washington University School of Medicine, St. Louis, MO, USA
203 Tohoku Medical Megabank Organization, Sendai, Japan
204 Department of Psychiatry, Washington University School of Medicine, St. Louis, MO, USA
205 Department of Public Health and Caring Sciences / Geriatrics, Uppsala University, Uppsala, Sweden
206 Epidemiology and Prevention Group, Center for Public Health Sciences, National Cancer Center, Tokyo, Japan
207 Department of Internal Medicine and the Center for Clinical and Translational Science, The Ohio State University, Columbus, OH, USA
208 Institute of Neuroscience and Physiology, the Sahlgrenska Academy at University of Gothenburg, Goteborg, Sweden
209 Department of Basic and Clinical Neurosciences, King's College London, London, UK
210 Department of Health Care Administration and Management, Graduate School of Medical Sciences, Kyushu University, Japan
211 Department of Medicine and Clinical Science, Graduate School of Medical Sciences, Kyushu University, Japan
212 Landspitali National University Hospital, Departments of Neurology & Radiology, Reykjavik, Iceland
213 Department of Neurology, Heidelberg University Hospital, Germany
214 Department of Neurology, Erasmus University Medical Center

215 Hospital Universitari Mutua Terrassa, Terrassa (Barcelona), Spain
216 Albert Einstein College of Medicine, Montefiore Medical Center, New York, NY, USA
217 John Hunter Hospital, Hunter Medical Research Institute and University of Newcastle, Newcastle, NSW, Australia
218 Centre for Prevention of Stroke and Dementia, Nuffield Department of Clinical Neurosciences, University of Oxford, UK
219 Department of Medical Sciences, Uppsala University, Uppsala, Sweden
220 Genetic and Genomic Epidemiology Unit, Wellcome Trust Centre for Human Genetics, University of Oxford, Oxford, UK
221 The Wellcome Trust Centre for Human Genetics, Oxford, UK
222 Beth Israel Deaconess Medical Center, Boston, MA, USA
223 Wake Forest School of Medicine, Wake Forest, NC, USA
224 Department of Neurology, University of Pittsburgh, Pittsburgh, PA, USA
225 BioBank Japan, Laboratory of Clinical Sequencing, Department of Computational biology and medical Sciences, Graduate school of Frontier Sciences, The University of Tokyo, Tokyo, Japan
226 Neurovascular Research Laboratory, Vall d'Hebron Institut of Research, Neurology and Medicine Departments-Universitat Autònoma de Barcelona. Vall d'Hebrón Hospital, Barcelona, Spain
227 Department of Biostatistics, University of Liverpool, Liverpool, UK
228 Wellcome Trust Centre for Human Genetics, University of Oxford, Oxford, UK
229 Institute of Genetic Epidemiology, Helmholtz Zentrum München - German Research Center for Environmental Health, Neuherberg, Germany
230 Department of Medicine I, Ludwig-Maximilians-Universität, Munich, Germany
231 DZHK (German Centre for Cardiovascular Research), partner site Munich Heart Alliance, Munich, Germany
232 Department of Cerebrovascular Diseases, Fondazione IRCCS Istituto Neurologico "Carlo Besta", Milano, Italy
233 Karolinska Institutet, MEB, Stockholm, Sweden
234 University of Tartu, Estonian Genome Center, Tartu, Estonia, Tartu, Estonia
235 Department of Clinical and Experimental Sciences, Neurology Clinic, University of Brescia, Italy
236 Translational Genomics Unit, Department of Oncology, IRCCS Istituto di Ricerche Farmacologiche Mario Negri, Milano, Italy
237 Department of Genetics, Microbiology and Statistics, University of Barcelona, Barcelona, Spain
238 Psychiatric Genetics Unit, Group of Psychiatry, Mental Health and Addictions, Vall d'Hebron Research Institute (VHIR), Universitat Autònoma de Barcelona, Biomedical Network Research Centre on Mental Health (CIBERSAM), Barcelona, Spain
239 Department of Neurology, IMIM-Hospital del Mar, and Universitat Autònoma de Barcelona, Spain
240 IMIM (Hospital del Mar Medical Research Institute), Barcelona, Spain
241 National Institute for Health Research Comprehensive Biomedical Research Centre, Guy's & St. Thomas' NHS Foundation Trust and King's College London, London, UK
242 Division of Health and Social Care Research, King's College London, London, UK
243 FIMM-Institute for Molecular Medicine Finland, Helsinki, Finland
244 THL-National Institute for Health and Welfare, Helsinki, Finland
245 Iwate Tohoku Medical Megabank Organization, Iwate Medical University, Iwate, Japan
246 BHF Glasgow Cardiovascular Research Centre, Faculty of Medicine, Glasgow, UK
247 deCODE Genetics/Amgen, Inc., Reykjavik, Iceland

248 Icelandic Heart Association, Reykjavik, Iceland
249 Institute of Biomedicine, the Sahlgrenska Academy at University of Gothenburg, Goteborg, Sweden
250 Department of Epidemiology, University of Maryland School of Medicine, Baltimore, MD, USA
251 Institute of Cardiovascular and Medical Sciences, Faculty of Medicine, University of Glasgow, Glasgow, UK
252 Chair of Genetic Epidemiology, IBE, Faculty of Medicine, LMU Munich, Germany
253 Division of Epidemiology and Prevention, Aichi Cancer Center Research Institute, Nagoya, Japan
254 Department of Epidemiology, Nagoya University Graduate School of Medicine, Nagoya, Japan
255 University Medicine Greifswald, Institute for Community Medicine, SHIP-KEF, Greifswald, Germany
256 Department of Neurology, Caen University Hospital, Caen, France
257 University of Caen Normandy, Caen, France
258 Department of Internal Medicine, Erasmus University Medical Center, Rotterdam, Netherlands
259 Landspitali University Hospital, Reykjavik, Iceland
260 Survey Research Center, University of Michigan, Ann Arbor, MI, USA
261 University of Virginia Department of Neurology, Charlottesville, VA, USA

Table S1. Description of summary data used in the analyses.

Traits	First authors /Consortia	Sample size	Population	Sex	Adjustments
Schizophrenia	Trubetskoy/PGC [13]	Case: 53, 386<br>Control: 77, 258	European	Mixed	Principal components
Ischemic stroke	Malik/MEGASTROKE [14]	Case: 34,217<br>Control: 406,111	European	Mixed	Principal components,<br>Sex, age
Large artery stroke	Malik/MEGASTROKE [14]	Case: 4,373<br>Control: 146,392	European	Mixed	Principal components,<br>Sex, age
Small vessel stroke	Malik/MEGASTROKE [14]	Case: 5,386<br>Control: 192,662	European	Mixed	Principal components,<br>Sex, age
Cardioembolic stroke	Malik/MEGASTROKE [14]	Case: 7,193<br>Control: 204,570	European	Mixed	Principal components,<br>Sex, age
Intracerebral hemorrhage	FinnGen [15]	Case: 1,687<br>Control: 201,146	European	Mixed	Principal components
Smoking initiation	Liu/GSCAN [24]	Case: 311,629<br>Control: 321,173	European	Mixed	Principal components

PGC, Psychiatric Genomics Consortium; GSCAN, GWAS and Sequencing Consortium of Alcohol and Nicotine use

Table S2. Heterogeneity test of inverse variance weighted fixed effect estimates.

	Q	P-value	I^2 index
Ischemic stroke	322.9	<0.000	23.8
Large artery stroke	323.9	<0.000	25.0
Small vessel stroke	254.9	0.319	3.9
Cardioembolic stroke	254.5	0.267	4.9
Intracerebral hemorrhage	267.0	0.139	9.0

Table S3. Multivariable Mendelian randomization analysis of the associations between schizophrenia and stroke subtypes including smoking initiation.

Outcomes	OR	95% CI	P-value	Q (P-value)
Ischemic stroke (365 SNPs)	1.009	0.984–1.035	0.485	459.1 (<0.000)
Large artery stroke (363 SNPs)	1.002	0.951–1.054	0.954	439.7 (0.002)
Small vessel stroke (364 SNPs)	1.043	0.989–1.100	0.125	385.0 (0.185)
Cardioembolic stroke (361 SNPs)	1.063	1.016–1.112	0.009	396.0 (0.081)
Intracerebral hemorrhage (361 SNPs)	1.070	0.984–1.164	0.114	446.7 (0.001)

OR, odds ratio; CI, confidence interval; SNPs, single nucleotide polymorphisms; MR, Mendelian randomization

Table S4. Results of leave one out analysis for the association between schizophrenia and intracerebral hemorrhage restricted to influential SNPs.

SNP	OR (95% CI)	P-value
rs12293670	1.08 (1.00–1.17)	0.053
rs2252074	1.08 (1.00–1.17)	0.052
rs2455415	1.08 (1.00–1.17)	0.051
rs2456020	1.08 (1.00–1.17)	0.052
rs72802868	1.08 (1.00–1.17)	0.050

SNP, single nucleotide polymorphism; OR, odds ratio; CI, confidence interval

Figure S1. Leave one out analysis for the association between schizophrenia and cardioembolic stroke using the inverse variance weighing method.

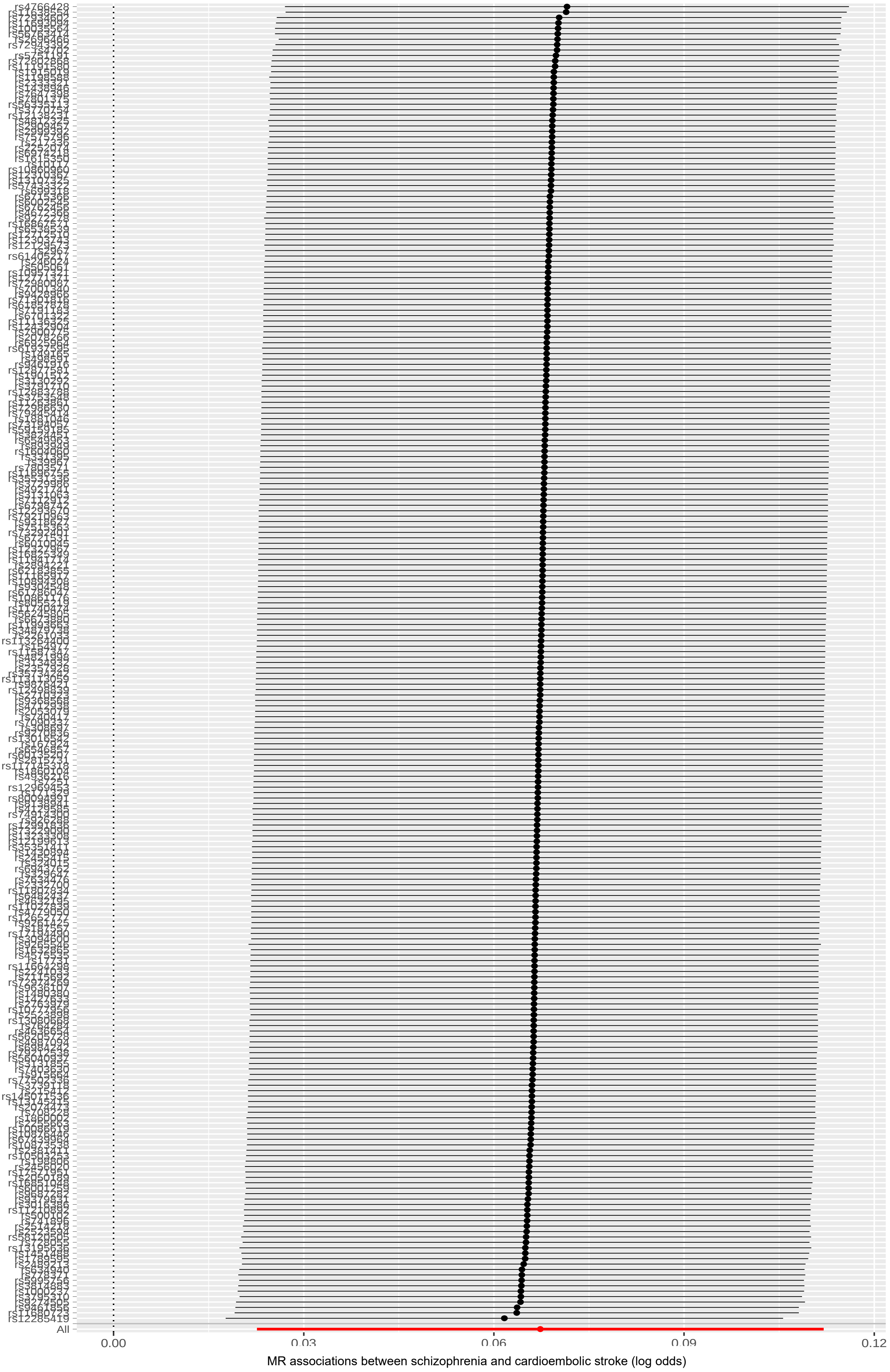

Figure S2. Leave one out analysis for the association between schizophrenia and intracerebral hemorrhage using the inverse variance weighing method.

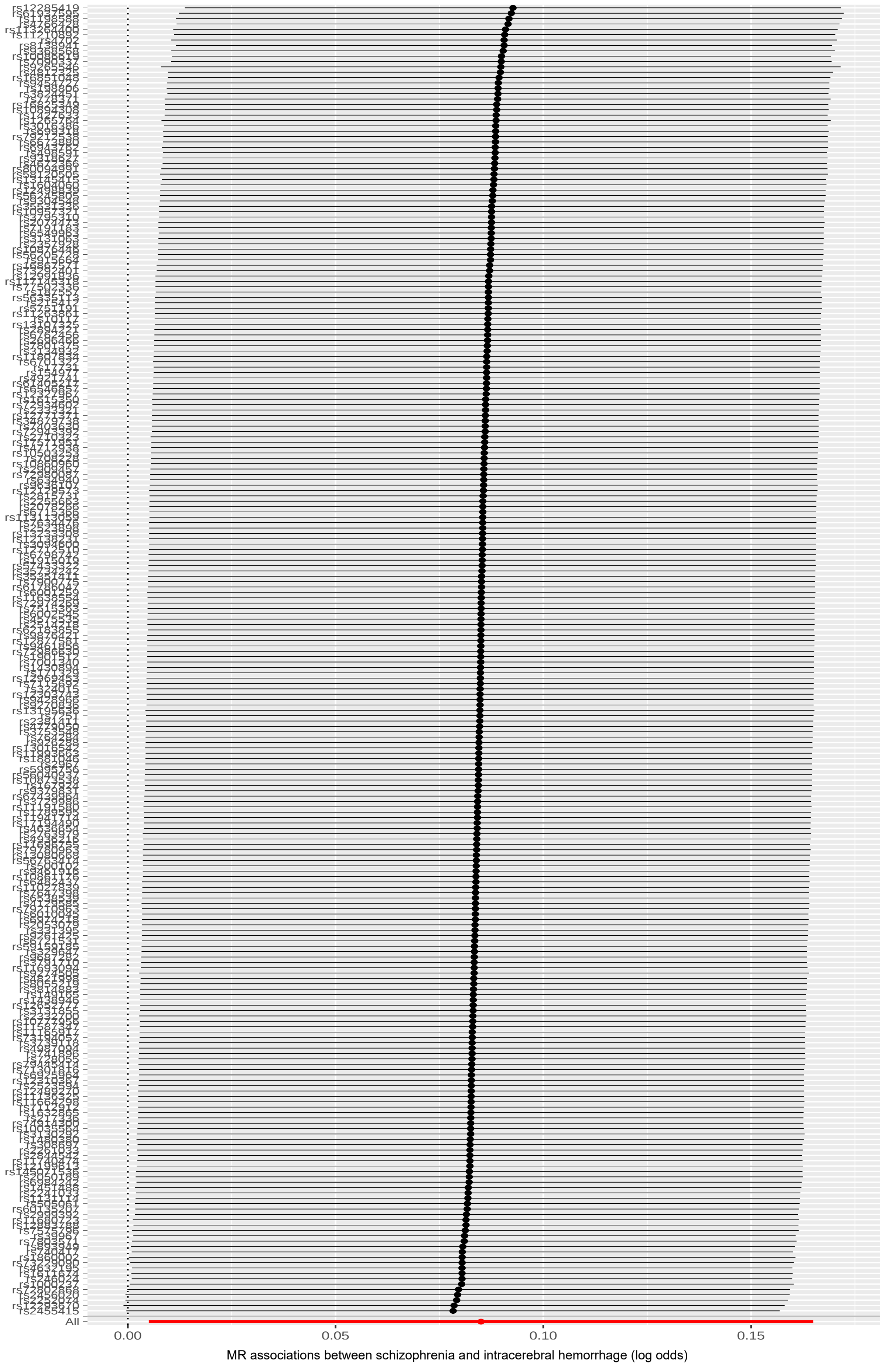

Supplement: Supplementary file 1 — Data S1 Tables S1–S4 Figures S1–S2 [file JAH3-13-e032011-s001.pdf]
